# Barriers to accessing perinatal mental health services and suggestions for improvement: qualitative study of women of Black and south Asian backgrounds

**DOI:** 10.1192/bjb.2024.82

**Published:** 2025-10

**Authors:** Nikolina Jovanović, Katy C. Packer, Mebh Conneely, Sarah Bicknell, Alex Copello, Rose McCabe, Ayşegül Dirik, Jelena Janković

**Affiliations:** 1 Queen Mary, University of London, London, UK; 2 East London NHS Foundation Trust, London, UK; 3 Camden and Islington NHS Foundation Trust, London, UK; 4 North East London NHS Foundation Trust, London, UK; 5 University College London, London, UK; 6 Birmingham and Solihull Mental Health Foundation Trust, Birmingham, UK; 7 University of Birmingham, Birmingham, UK; 8 City, University of London, London, UK

**Keywords:** Perinatal, psychiatry, mental health services, access, barriers, ethnic and racial minorities

## Abstract

**Background:**

Maternity outcomes for women from certain ethnic groups are notably poor, partly owing to their not receiving treatment from services.

**Aims:**

To explore barriers to access among Black and south Asian women with perinatal mental health problems who did not access perinatal mental health services and suggestions for improvements, and to map findings on to the perinatal care pathway.

**Method:**

Semi-structured interviews were conducted in 2020 and 2021 in the UK. Data were analysed using the framework method.

**Results:**

Twenty-three women were interviewed, and various barriers were identified, including limited awareness of services, fear of child removal, stigma and unresponsiveness of perinatal mental health services. Whereas most barriers were related to access, fear of child removal, remote appointments and mask-wearing during COVID-19 affected the whole pathway. Recommendations include service promotion, screening and enhanced cultural understanding.

**Conclusions:**

Women in this study, an underrepresented population in published literature, face societal, cultural, organisational and individual barriers that affect different aspects of the perinatal pathway.

People from ethnic minority backgrounds face higher risks of perinatal mental health problems^1–4^ and are less likely to receive appropriate treatment.^5–7^ In the UK, individuals from Black and south Asian ethnic groups have increased likelihood of experiencing adverse maternity outcomes, including a 2–4 times higher risk of maternal death.^8^ In addition to the barriers that affect all parents in accessing mental health services, such as stigma, service fragmentation and fear of child removal,^9^ individuals from ethnic minority groups face further challenges. These include language barriers, lack of confidence in navigating services and discrimination within healthcare settings.^10–12^ No research has yet explored experiences of women who did not access mental health services despite suffering from perinatal mental ill health. These women are likely to face increased difficulties in accessing support owing to their multiple stigmatised and marginalised identities.^13^ In the UK, substantial investments have been made in specialist perinatal mental health services (PMHS) so that women with moderate to severe mental illness can receive evidence-based treatments promptly.^14–16^ The current study explored the perceived barriers to accessing PMHS among women from Black and south Asian backgrounds who had experienced perinatal mental ill health and also did not get help from services. In addition, the study explored women's recommendations for improving access to PMHS. Last, the identified barriers and recommendations were mapped onto the perinatal care pathway to enhance our understanding of how they affect various stages of the pathway.

## Method

### Study design

This qualitative study was conducted during January 2020 and August 2021 in the UK. The study used semi-structured interviews to gather data from participants, who were recruited from through media platforms, third-sector organisations and clinical services. The study adhered to the COREQ checklist and was approved by the Health Research Authority through the Research Ethics Committee (reference: 19LO1830). The study protocol can be accessed on the Open Science Framework (https://osf.io/s94bp/).

### Participants and procedure

The eligibility criteria include: (a) self-identification as a Black or south Asian woman; (b) age over 16 years; (c) active involvement in the care of their baby; (d) self-reported or diagnosed moderate to severe perinatal mental health difficulties within the past 2 years; (e) no utilisation of PMHS; and (f) willingness and capacity to provide informed consent.

None of the study participants had ever utilised PMHS. This was either because they were not referred to the service, declined the referral or did not attend appointments. In addition, women who attended triage or the first assessment but did not attend any subsequent appointments were also eligible for inclusion in the study. Women who were referred to PMHS were recruited through East London NHS Foundation Trust and Birmingham and Solihull Mental Health Foundation Trust. Women who declined a referral to PMHS or were never referred were recruited through a social media advertisement and community organisations that provide support to women facing perinatal mental ill health. A member of the research team contacted potential participants. Snowball sampling was also used, starting from the participants who were already interviewed. During the study period, self-referrals to PMHS were not available. Supplementary Material 1 available at https://doi.org/10.1192/bjb.2024.82 shows the recruitment pathways.

Interviews were conducted over the phone or using video call by three researchers (K.C.P., S.B. and A.S.), one of whom has lived experience of perinatal mental illness. Although the study protocol planned for face-to-face interviews, the transition to remote interviewing was needed owing to the COVID-19 pandemic. Before the interview, the researcher provided participants with an information sheet, gained informed consent and completed a demographic information form. All study participants provided verbal consent to participate in the study; this was audio/video recorded. Participants were reimbursed for their time with a £30 shopping voucher. Interviews were audio recorded and transcribed verbatim by an external transcription agency.

### Materials

An interview guide was developed by the research team and the Lived Experience Advisory Panel (LEAP). It explored women's experience of living with perinatal mental ill health, the impact of their illness on their relationships, support from family and friends, barriers to accessing PMHS, perceptions of PMHS, suggestions to improve the accessibility of PMHS, use of non-NHS (National Health Service) services and how women managed their illness without treatment. The interview guide is included in Supplementary Material 2.

### Data analysis

Data were analysed using framework analysis, a systematic matrix-based method with five distinct steps.^17^ Two researchers (K.C.P. and S.B.) met regularly to review the developing emergent themes. The themes were regularly reviewed in discussion with the wider analysis team (N.J., J.J., M.C., A.C. and R.M.) and with the LEAP.

## Results

### Study participants

All study participants were women who identified their gender as female. The study included 23 women, with 16 from south Asian backgrounds and seven from Black ethnic backgrounds. Two recruited participants did not proceed with the interviews owing to busy schedules. In total, 16 of the women were never referred to PMHS, and seven declined their referral to PMHS or did not attend their appointments. The interview lengths ranged from 13.36 min to 126 min (mean = 46.91 min). The participants’ characteristics, including ethnicity and self-reported mental health disorders, are presented in Table 1.

**Table 1:** Study participants (*n* = 23)

Characteristics		*n* (%)
Age range, years	20–29	4 (17)
	30–39	17 (74)
	40–49	2 (9)
Country of birth	UK	16 (70)
	Outside UK	7 (30)
Ethnicity	Asian Indian	7 (30)
	Asian Bangladeshi	4 (17)
	Asian Pakistani	4 (17)
	Black Caribbean	3 (13)
	Black African	2 (9)
	Asian Mauritian	1 (4)
	White and Black Caribbean	1 (4)
	Other	1 (4)
Religion	Muslim	9 (3)
	Christian	8 (35)
	Sikh	4 (17)
	Seventh Day Adventist	1 (4)
	No religion	1 (4)
Level of education	GCSE and A-level	2 (9)
	Vocational education	4 (17)
	Bachelor's degree	13 (57)
	Postgraduate degree	4 (17)
Relationship status	Married or cohabiting	17 (74)
	Partner but not cohabiting	4 (17)
	Separated or divorced	2 (9)
Number of children	1	4 (17)
	2	12 (52)
	3	6 (26)
	4	1 (4)
	5	4 (17)
Self-reported mental health difficulties and/or diagnoses^a^	Depression/postnatal depression	14 (61)
	Anxiety	12 (52)
	Post-traumatic stress disorder	4 (17)
	Stress	2 (9)
	No diagnosis	2 (9)
	Not known	1 (4)

a. Several participants reported more than one diagnosis.

### Barriers to accessing PMHS

Three themes related to perceived barriers were identified, each consisting of several subthemes (Table 2). Themes, subthemes and illustrative quotes from study participants are summarised in Supplementary Material 3.

**Table 2:** Barriers to access and suggestions for improvement – overview of study findings

Barriers to access	
Theme 1: Barriers resulting from societal and cultural factors	
Subthemes	1.1. Fear and shame of being judged as a failure as a mum
	1.2. Mental health is a taboo subject in some Asian and Black communities
	1.3. Seeking help from professionals is viewed as being weak as a person
	1.4. No privacy in intergenerational homes
	1.5. Negative treatment of ethnic minority women and cultural insensitivity in healthcare services
Theme 2: Barriers resulting from women's understanding and perceptions of PMHS	
Subthemes	2.1. Unaware PMHS exist
	2.2. Fear of having their baby removed
	2.3. Minimising perinatal mental health symptoms
	2.4. Negative perceptions of health services
	2.5. Lack of financial and time resources
Theme 3: Barriers resulting from the organisation and structure of healthcare services	
Subthemes	3.1. Poor response from PMHS (only participants referred to PMHS)
	3.2. Healthcare professionals did not offer a referral to PMHS
	3.3. No opportunity to discuss mental health with healthcare professionals
	3.4. Harder to open up when no continuity with healthcare professionals
	3.5. Harder to seek support during COVID-19 pandemic
Theme 4: Suggestions for improvement	
Subthemes	4.1. All mothers should be asked about their mental health
	4.2. Improvements related to primary care
	4.3. Increasing awareness of PMHS and sharing that the goal is to keep mother and baby together
	4.4. Increased understanding of different ethnicities and cultures amongst healthcare professionals
	4.5. Support groups for mums from ethnic minority backgrounds

PMHS, (community) perinatal mental health services.

Barriers arising from societal and cultural factors (theme 1) included women's overall fears of being perceived as inadequate mothers, mental health stigma, and negative encounters with healthcare services when dealing with women from ethnic minority backgrounds. In addition, some south Asian women living in intergenerational homes lacked the privacy to receive calls, post or visits from PMHS, which was a barrier to accessing and engaging with services. Study participants often described these factors operating together simultaneously.

Not knowing about the existence and offerings of services and negative perceptions of PMHS (theme 2), also acted as access barriers. The prominent fear expressed by our participants was that services would remove the baby from the mother's care. Women minimised their symptoms in front of healthcare professionals and chose to rely exclusively on family support. Insufficient financial and time resources were also significant barriers to attending appointments.

The third group of barriers resulted from the organisation of healthcare services (theme 3). Women who were referred to PMHS found the services non-responsive; they were either never contacted by PMHS, or the response was too slow. Those who were not referred felt that clinicians failed to make the referral although women disclosed mental health difficulties. Others were not given the opportunity to disclose their difficulties or struggled to engage owing to lack of continuity of care. Remote working and wearing masks during appointments were also seen as barriers to engagement.

### Recommendations to improve utilisation of PMHS

The participants suggested various ways to improve access to and engagement with PMHS for Black and south Asian women (Table 2). These included proactive inquiries about mental health for all mothers; improvements within primary care, as it serves as the initial point of contact with services for many women; increased promotion of services; better understanding of cultural perspectives on mental health among staff; and the establishment of support groups. The participants strongly emphasised that services need to share that their goal is to keep mother and baby together, rather than separating them, and dispelling the misconception that having a mental illness will lead to the removal of babies. This theme, subthemes and illustrative quotes are summarised in Supplementary material 4.

### Mapping key barriers and recommendations onto the pathway to PMHS

**Fig. 1:**
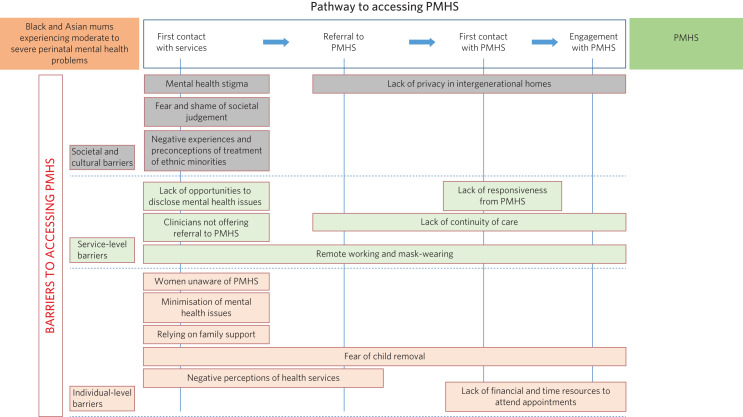
Key barriers mapped along the pathway to accessing community perinatal mental health services (PMHS).

Figure 1 illustrates the pathway to PMHS, which starts with the first patient contact with health services. Subsequently, a referral to PMHS is made, and, upon acceptance, the service offers the first appointment within 28 days. Engagement with PMHS is defined as attending the initial and subsequent appointments.

Key barriers to accessing PMHS can be grouped into societal and cultural, service/organisational and individual barriers. As depicted in Fig. 1, a total of 11 barriers were mapped at the stage of first contact with health services, hindering the disclosure and recognition of mental health issues. These barriers had a cascading effect on the subsequent parts of the pathway. Two barriers had a pervasive impact on all parts of the pathway: the fear of child removal by services, and COVID-19 restrictions. One specific cultural factor, namely lack of privacy in some intergenerational south Asian families, made it difficult for women to agree to the referral and to engage with PMHS. The point of making referral to PMHS was affected by two additional barriers: lack of continuity of care and negative perception of services. Lack of financial and time resources was an important barrier to attending appointments with PMHS.

**Fig. 2:**
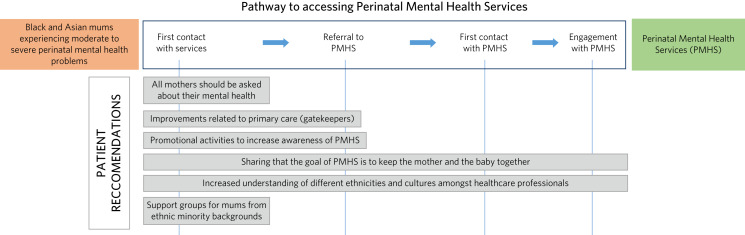
Study participants’ recommendations integrated within the perinatal pathway.

Figure 2 depicts the integration of participants’ recommendations within the perinatal pathway. Various recommendations, such as inquiring about mental health, enhancing primary care practices and promoting PMHS on a broader scale, have the potential to improve service accessibility. Overcoming two specific barriers – namely, communicating the purpose of services as support for mothers and infants, and increasing staff awareness of diverse ethnicities and cultures – could have a positive impact on every stage of the pathway. In addition, the provision of support groups for mothers who prefer not to access services could prove beneficial, potentially facilitating their engagement with specialist interventions if necessary.

## Discussion

### Main findings

This study is the first to explore the perspectives of women who did not utilise PMHS despite grappling with mental health issues during pregnancy and after childbirth. Three main findings stand out. First, the identified barriers resulted from societal and cultural factors, service organisation factors, and individual factors. Although most of these barriers were associated with access, three barriers – fear of child removal, remote appointments and mask-wearing during COVID-19 – had pervasive effects throughout the entire perinatal pathway. Second, these barriers did not exist in isolation; rather, the participants described multiple examples of complex interplay between them. Last, efforts to address these barriers could include increasing awareness of PMHS, advertising that the goal of services is to keep the mother and baby together, universal maternal screening, staff training, streamlining referrals from primary care, and setting up support groups for parents from the same ethnic minority background. These are actionable recommendations for improving access and utilisation of services.

### Comparison with the literature

The issue of non-engagement or disengagement has immense significance in mental healthcare because it may lead to worse outcomes and inefficient use of resources.^18^ Therefore, it is extremely important that voices of people not accessing services are explored and reported.

This study identified several barriers that have already been identified in published research, including stigma, fear of child removal, negative experiences and preconceptions of treatment in different cultures, lack of opportunities to disclose mental health issues, and negative perceptions of services.^11–14^ Watson and colleagues argued that women from ethnic minority backgrounds face ‘double stigma’ or the intersectionality of experiencing prejudice and discrimination in healthcare settings on top of the stigma attached to having mental health problems.^13^ This is supported by the current findings. Women expressed concerns about being stereotyped because of their ethnicity or anticipated that services would be facilitated by White healthcare professionals who would lack the cultural understanding and sensitivity to provide appropriate care. This affected how likely women were to disclose their mental health problems and in some cases deterred them from accessing support altogether.

Importantly, this study also identified barriers that may have unique significance for ethnic minority women who do not access or engage with services. These barriers include a lack of awareness of available services (especially relevant for immigrants), the tendency to minimise mental health problems and rely on family support, insufficient privacy in multigenerational south Asian families to accept home visits and receive clinical letters, COVID-19 restrictions, and a lack of financial and time resources to attend appointments.

For instance, women consistently expressed their lack of knowledge about PMHS and their limited understanding of the services’ scope. This can be partly attributed to the relatively recent establishment of these services within the healthcare system.^1^ However, it is noteworthy that PMHS have been undergoing significant expansion for several years before this study. Despite ongoing efforts, it appears that at the time of the study, these services were not sufficiently known to relevant clinicians who could make referrals (e.g. general practitioners, midwives and health visitors) or to patients. Our participants mentioned instances where clinicians did not offer referrals to PMHS, even after the women disclosed their perinatal mental health issues. In our previous publication, patients reported that their journey to accessing services was complex and unpredictable, as they stumbled upon these services by chance.^19^ One of the recommendations put forward by study participants was to focus on the promotion and advertisement of services. However, it is important to note that promotion efforts need to be carefully planned to ensure that increased interest in services does not overwhelm the capacity to meet a surge in referrals from professionals and self-referrals.

Another significant barrier at the service level was the lack of responsiveness from PMHS during the initial contact phase. It is important to acknowledge that services are given a 28-day window to respond to referrals and establish first contact with the patient.^20^ Whereas this may be seen as an appropriate timeframe from a service perspective, it may appear too long from the perspective of a mother experiencing mental health issues. To enhance communication from PMHS when referrals are made, it would be beneficial to provide transparent information about waiting times once women have been referred. This transparency could help prevent women from feeling frustrated or overlooked. Early contacts with patients could explore their cultural background and attempt to work around stigma and limitations within family and support networks. It is also important to prioritise continuity of care and avoid overwhelming women with excessive contacts and appointments, which can easily occur during the perinatal period.

As mentioned, the fear of child removal is a longstanding barrier that continues to hinder access to services, and its impact was observed across all stages of the perinatal pathway. Addressing this significant barrier requires a multi-faceted approach from various sources. Participants in this study emphasised the importance of promoting services by highlighting the role of PMHS in supporting mothers and infants, facilitating the development of a healthy mother–infant bond, and assisting them in establishing or strengthening their existing support networks. This aspect could be instrumental in alleviating the fear of child removal and fostering a sense of trust and assurance among potential patients.

Our participants emphasised that certain barriers were specifically associated with COVID-19 restrictions, such as remote appointments and mask-wearing. These factors posed challenges for women in feeling comfortable to disclose their mental health difficulties. These findings contribute to our understanding of why women from ethnic minority groups may have been disproportionately affected during the COVID-19 pandemic.^21^ It is crucial to acknowledge and address these barriers, as remote appointments continue to be prevalent even in the post-COVID era. For example, in the UK, approximately one-third of clinical appointments within primary care are still conducted remotely.^22^

It is clear that the recommendations for improvement identified in this study align with established best practices and interventions for which benefits have already been demonstrated. The fact that these recommendations are not yet widely implemented highlights challenges in the implementation process. The translation of evidence into clinical care continues to be a significant issue in healthcare, effectively hindering patients from benefiting from innovative and evidence-based practices.^23^

### Strengths and limitations

This study amplified the voices of a particularly vulnerable group of women and offered valuable insights into their healthcare experiences, thereby filling a gap in the published literature. The research was guided by the LEAP, which included individuals who had personal experience with perinatal mental illness and belonged to minoritised ethnicities. In addition, the study benefited from the diversity of the professional and ethnic backgrounds of its team members.

This study had several limitations. The categorisation of groups based on ethnicity may lead to the assumption that individuals within each group are homogeneous, which is rarely true. Next, while the study employed various strategies to recruit women who did not access or engage with services, it did not allow for the inclusion of non-English-speaking women. It is likely that these women face additional barriers when accessing services that were not captured in our current sample. Another limitation is that the severity of mental health problems was based on self-report. It is possible that the perinatal mental health problems experienced by women in our study were not severe enough to meet the criteria for support from PMHS, which are typically commissioned to treat women with moderate to severe mental illness. However, it is important to note that the women interviewed expressed significant distress, with many describing suicidal ideation, suggesting that they would probably have benefited from specialist interventions.

In addition, we acknowledge that owing to the COVID-19 pandemic, interviews were conducted remotely, which may have made it challenging for researchers to pick up on non-verbal cues and build rapport with participants. However, remote interviewing also had its benefits, such as allowing for the continuation of research during the pandemic, expanding recruitment across England and enabling participation from individuals who may have otherwise faced transport barriers. It is also possible that some participants felt more comfortable opening up about sensitive issues during telephone calls.

Last, it is worth highlighting that a higher proportion of south Asian women (69%) were recruited for this study compared with Black women (22%). When considering the UK 2021 census data, which reported a population of 5.4 million south Asian individuals (9.6%) and 2.4 million Black individuals (4.2%), it becomes evident that the representation of Black women in the study was not sufficient. This aligns with the well-established marginalisation of Black women, even within research that focuses on marginalised groups.^24,25^

### Implications of the findings for practice and research

This study has uncovered various cultural, organisational and individual barriers that hinder access to PMHS for women of Black and south Asian ethnic backgrounds in the UK. Furthermore, the study's findings shed light on how these barriers affect different parts of the perinatal care pathway. The timing of this research is significant as it coincides with a global increase in awareness surrounding perinatal mental health concerns. Governments worldwide are striving to achieve the UN Sustainable Development Goals aimed at improving health outcomes for mothers, infants, children and families. In the UK, PMHS continue to expand, allowing for greater access to specialised care and treatment for women in both community and in-patient settings. This research offers valuable insights that could assist policy makers and service providers in enhancing access for Black and south Asian women. These groups are less likely to receive support from services and were disproportionately affected by the COVID-19 pandemic. Through implementation of changes based on these insights, services could be made more accessible to all women who could benefit from specialist treatment and care. For future research, we recommend concentrating on addressing the identified barriers, employing innovative recruitment strategies to ensure the inclusion of underrepresented groups and investigating the reasons behind the limited implementation of certain recommended practices, despite their being considered best practices.

## Supporting information

Jovanović et al. supplementary material Jovanović et al. supplementary material

## Data Availability

The data that support the findings of this study are available on request from the corresponding author.

## About the authors

**Nikolina Jovanović** is Reader in Perinatal Psychiatry and a consultant psychiatrist at the Centre for Psychiatry and Mental Health, Wolfson Institute of Population Health, Queen Mary, University of London, London, UK; and East London NHS Foundation Trust, London, UK. **Katy C. Packer** is a trainee psychologist at East London NHS Foundation Trust, London, UK; Camden and Islington NHS Foundation Trust, London, UK; and North East London NHS Foundation Trust, London, UK. **Mebh Conneely** is a research fellow at the Centre for Psychiatry and Mental Health, Wolfson Institute of Population Health, Queen Mary, University of London, London, UK; East London NHS Foundation Trust, London, UK; and the Division of Psychiatry, University College London, London, UK. **Sarah Bicknell** is Senior Research Fellow at Birmingham and Solihull Mental Health Foundation Trust, Birmingham, UK. **Alex Copello** is Professor of Addiction Research and a consultant clinical psychologist at Birmingham and Solihull Mental Health Foundation Trust, Birmingham, UK; and the School of Psychology, University of Birmingham, Birmingham, UK. **Rose McCabe** is Professor of Clinical Communication at the School of Health and Psychological Sciences, City, University of London, London, UK. **Ayşegül Dirik** is a research fellow at the Centre for Psychiatry and Mental Health, Wolfson Institute of Population Health, Queen Mary, University of London, London, UK; and East London NHS Foundation Trust, London, UK. **Jelena Janković** is a consultant perinatal psychiatrist at Birmingham and Solihull Mental Health Foundation Trust, Birmingham, UK.
