# Supplementary material for: Barriers to accessing perinatal mental health services and suggestions for improvement: qualitative study of women of Black and south Asian backgrounds
Source: BJPsych Bull. 2025 Oct;49(5):299–306. doi: 10.1192/bjb.2024.82 (PMC12501512; doi:10.1192/bjb.2024.82)
Supplement: Jovanović et al. supplementary material [file S2056469424000822sup001.docx]

**Supplementary material 1**

# Study recruitment pathways

Snowballing

(*n*= 1)

Community Organisations

(*n*= 6)

Social Media

(*n*= 9)

NHS services

(*n*= 6)

Social Media

(*n*= 1)

Referred to PMHS, but did not engage (*n*= 7)

Participants interviewed

(*n*= 23)

Not referred to PMHS

(*n*= 16)

## Supplementary material 2 - Interview guide

**Interview guide: Qualitative interviews for PAAM (WP3):**

**Mums with PMI that have NOT accessed PMHS**

1. **Introduction**

- Introduce self
- Explain nature and purpose of the research (e.g. this interview is about exploring your experience of perinatal mental health illness, and how we can make perinatal mental health services better and easier to use for mums from ethnic minorities)
- Provide assurances about confidentiality and no link to current treatment/care (e.g. your name and information will only be known by the researchers and will not be revealed to anyone else)
- Explain what happens to data collected – recording, transcribing, analysis, anonymity (all potentially identifying information will be removed), audio recordings will be destroyed once data analysis is complete, check how they would like to be referred to during recording
- Introduce tape recorder
- Explain how interview works- interviewer will not say very much and will tend to ask questions, all views important, no right or wrong answers, looking for a range of views
- To say if they are feeling stressed/uncomfortable. Do not have to answer a question or can change topic if needed. The interview can be paused at any time if you require a break.
- Mobile phones off or on silent
- Invite any questions

*Note for interviewer:*

*As far as possible make sure you probe and ask for specific examples. So, if the participant states that ‘needs specific to their culture were not met’ ask the participant to provide specific examples of this.*

1. **Background**

**🡪 *Ask participant about themselves (e.g. how many children they have, who they live with, do they work, availability of close friends and family members as support) and about their experience of pregnancy/ies and postnatal period,***

*e.g. tell us a bit about yourself and your experience of pregnancy and after the baby was born*

*The purpose of this section is to obtain a clear understanding of the mother’s socio economic context and experience of pregnancy/ies and postnatal period (not focusing specifically on mental health problems- that is covered in the next section)*

*By the end of this section the interviewer should have a clear understanding of the mother’s mental health difficulties, including the origin, early signs, development and impacts on mother and child as well as the wider family if relevant.*

***Map out previous pregnancies and associated mental health problems***

*e.g. tell us a bit about your experience of mental health problems in pregnancy and after the baby was born*

- *Ask participant about:*
  - *Their experience of mental health problems (such as feeling low, worried, difficulties bonding with a baby, unusual experiences/thoughts)*
    - *During pregnancy*
    - *After the baby was born*
  - *(If relevant) In this interview, I will mostly be asking you about your experiences of your most recent pregnancy/childbirth (1-2 years), but please feel free to share with me your previous experiences.*

Potential probes:

- When did these problems start?
- What is your own understanding of this and is there anything that you think may have caused it?

1. **Dealing with perinatal mental illness**

🡪 ***Ask participant about how they coped with perinatal mental illness***

**Experiences of receiving help from non-PMHS**

Now I would like to talk to you about the care you received for your perinatal mental illness

- Did you or your family feel that you needed some help for your perinatal mental illness?
  - If yes from whom and what was that experience like?
    - What was helpful / less helpful?
    - What was good / not good?
  - If not – from your experience what were the factors that prevented you from seeking help?
  - What might have encouraged/helped you to seek help for your difficulties?
- How did you/your family feel about you seeking help?

**Acceptability of non-PMHS (culture/ethnicity)**

- In your opinion, has your culture/ethnicity been taken into consideration in the support you received? Can you give me examples of this?
- Do you think they meet the needs of a South Asian/Black woman like yourself?
- Considering your ethnic background, was there anything important missing from your care?

**Understanding reasons for non-engagement with PMHS (IF RELEVANT)**

*By the end of this section, the interviewer should have an understanding of the reasons for non-engagement with the services and whether cultural/ethnic factors influenced this*

- Now I would like to explore your experiences of being referred to PMHS
  - What were your initial thoughts about being referred?
  - Did you know about PMHS?
  - What kind of support did you expect to receive from the service?

Did you have any worries or concerns about a referral?

- If a referral to PMHS was suggested, were there any reasons that prevented you from attending your appointments? SPONTANEOUS, THEN PROMPT:
  - Language
  - Childcare
  - Location
  - Stigma
  - Fear
- Is there something that could have made it easier for you to attend your appointments with the PMHS? How do you think PMHS could have helped with your mental health illness and the difficulties you were facing? How do you think care offered to mums from ethnic minorities with perinatal mental illness could be improved?

**End of interview**

- Any final thoughts or ideas – anything they would like to add
- Thank participants for their time
- Remind re: Confidentiality and recording
- Reimbursements of £

**Supplementary material 3**

**Perceived barriers to accessing perinatal mental health services – themes, subthemes, and participants’ quotes**

| Theme 1: Barriers resulting from societal and cultural factors | |
| --- | --- |
| Subthemes | *Participants’ quotes* |
| - 1. Fear and shame of being judged as a failure as a mum | *Saying […] I’m feeling overwhelmed by just looking after the baby, whatever responsibilities I had, was a sign of failure […] So, I was kind of scared and ashamed to admit that. (Participant 17, Black African)* |
| - 1. Mental health is taboo subject in some Asian and Black communities | *[…] Mental health is very much opposed, it’s not really a topic, it’s just nothing that we ever spoke about in my house. […] We taught so many things […] about even just physical health. […] But never […] conversations about how you feeling. (Participant 5, Black Caribbean)* |
| - 1. Seeking help from professionals is viewed as being weak as a person | *Because in our [Asian] culture the lady doesn’t seek any medical advice from professionals, they just think that we should overcome ourselves all around. […] I thought that for long a time I had to look after myself and my son, […] I should have make myself strong. (Participant 12, Asian Indian)* |
| - 1. No privacy in intergenerational homes | *I think it’s also harder for women who live with extended families, as most Asians do. […] You constantly feel like you’re being watched. Whereas it’s easier to access if you’re living on your own, away from your in-laws because post comes to your house, if somebody phones you have the privacy that you need. (Participant 9, Asian Pakistani)* |
| - 1. Negative treatment of ethnic minority women and cultural insensitivity in healthcare services | *Asian families can have quite long last names […] [when] my cousin was thinking about naming her child, one of the midwives said, oh make sure you don’t name their first name as something long because your last name is too long, […] So, I think you maybe get some of those kind of comments, [so]maybe you do feel more guarded (Participant 7, Asian Mauritian)* |
| Theme 2: Barriers resulting from women's understanding and perceptions of PMHS | |
| 2.1. Unaware PMHS exist | *I feel like I’ve heard the term, but I wouldn't be able to tell you anything about it****.***  *[…] I wouldn't ever have thought that you could go [to MBU] with the baby. I thought you'd have to go on your own. And I wouldn't be able to see [the baby] (Participant 11, Black Caribbean)* |
| 2.2. Fear of having their baby removed | *I did feel like I really wanted to talk to someone […], but I was like well hang on a minute if I thought if I start opening up, then she [midwife] might take my baby away and that’s the gods honest truth. Because she did ask me about it and I pushed it off and I just said that yeah, everything's fine. (Participant 14, Asian Indian)* |
| 2.3. Minimising perinatal mental health symptoms | *I just wanted to almost fool myself as well just pretending that I was ok and everything was fine.  As long as [Son’s name] was ok, I’m ok, which is, like I said in hindsight, definitely wasn’t the right approach and that would’ve been a good opportunity to, to seek help as well. (Participant 18, Asian Indian)* |
| 2.4. Negative perceptions of health services | *If it's coming across clinical, then no, I don't want to attend because I've got plenty of appointments that went on during pregnancy. I don't need another appointment and you know, go into a hospital atmosphere, just horrible […] (Participant 16, Asian Bangladeshi)* |
| 2.5 Lack of financial and time resources | *It’s also to do with finances to be fair […] imagine having to take time off or asking my partner to stay off work so that I could get help. The longer he stays out, off work we have bills, we have families to look after. (Participant 17, Black African)* |
| Theme 3: Barriers resulting from the organisation and structure of healthcare services | |
| 3.1. Poor response from PMHS  (*Only participants referred to PMHS)* | *I needed them [the NHS] to be there for me and they failed me […] I thought people often don’t get help because they don't ask for help. […] you could sit there and scream mental health but if you don’t have the right person [to realise you need help], you won't get it. (Participant 1, Asian Pakistani)* |
| 3.2. Healthcare professionals did not offer a referral to PMHS | *I was still feeling low [when they discharged me] […] If they, at that precise moment in time, if they had said, you can get help from, if you’re still feeling low, call this number, you can get help from this place or that place then I probably would have. (Participant 20, Black African).* |
| 3.3. No opportunity to discuss mental health with healthcare professionals | *[During pregnancy] I think if someone had had a frank conversation with me, professionals, I think I would have admitted it, but no one ever had those kind of conversations with me. […] I would have honestly, I would have answered, yes, I am struggling. (Participant 19, Asian Indian)* |
| 3.4. Harder to open up when no continuity with healthcare professionals | *My health visitors were changing all the time as well because of the constraints of the NHS. So that didn’t help as well because if it was a new person, I didn’t want wanna open up to them. […] Only after seeing them for two or three sessions that, erm they, they kind of, I became comfortable with them. (Participant 8, Asian Pakistani)* |
| 3.5. Harder to seek support during COVID-19 pandemic | *That's a really weird thing when you're trying to open up to somebody, but you both have a mask on. And you can read their expressions, and we were sat so far away. And so, I just, yeah, it just all felt really alien in that room. (Participant 11, Black Caribbean)* |

**Supplementary material 4**

**Study participants’ suggestions to improve access to PMHS for Black and South Asian women**

| Suggestions for improvements | *Participants’ quotes* |
| --- | --- |
| All mothers should be asked about their mental health | *You should be asking them [women] at every possible opportunity about their mental health as you’re asking them about their physical health. So, their physical wellbeing it’s, you know, is you ask them if the baby’s moving well why can’t you ask them if they’re, you know, how are you feeling and are you OK?  (Participant 19, Asian Indian)* |
| Improvements related to primary care | *[…] we do go through a GP referral it is really long, you know, time is really important for mums and parents. So, if it’s easily accessible, easy and quick, then maybe that might encourage mums to access it. (Participant 16, Asian Bangladeshi)* |
| Increasing awareness of PMHS and sharing that the goal is to keep mother and baby together | *If that's kind of their [PMHS] aim, like you said that the aim is that mum and baby stayed together, I think that needs to be broadcast. Because I can imagine, and I'm sure that I’m not the only one who would shy away from something like that. (Participant 11, Black Caribbean)* |
| Increased understanding of different ethnicities and cultures amongst healthcare professionals | *One-day training that’s not enough to get to know a particular ethnic minority or background you need to read up or spend time with them. (Participant 4, Asian Bangladeshi)* |
| Support groups for mums from ethnic minority backgrounds | *I would definitely be more receptive to an NHS based psychoeducational group where you can talk about your experiences and then to be in a group full of women, you know, a room full of women that’s, it could be quite empowering as well, because they’re South Asian too, and work through some of those myths. (Participant 13, Asian Indian)* |
